# Evidence for link between modelled trends in Antarctic sea ice and underestimated westerly wind changes

**DOI:** 10.1038/ncomms10409

**Published:** 2016-02-04

**Authors:** Ariaan Purich, Wenju Cai, Matthew H. England, Tim Cowan

**Affiliations:** 1 CSIRO Oceans and Atmosphere, Aspendale, Victoria 3195, Australia; 2 Climate Change Research Centre, University of New South Wales, Sydney, New South Wales 2052, Australia; 3 ARC Centre of Excellence for Climate System Science, University of New South Wales, Sydney, New South Wales 2052, Australia; 4 School of GeoSciences, University of Edinburgh, Edinburgh EH9 3FE, UK

## Abstract

Despite global warming, total Antarctic sea ice coverage increased over 1979–2013. However, the majority of Coupled Model Intercomparison Project phase 5 models simulate a decline. Mechanisms causing this discrepancy have so far remained elusive. Here we show that weaker trends in the intensification of the Southern Hemisphere westerly wind jet simulated by the models may contribute to this disparity. During austral summer, a strengthened jet leads to increased upwelling of cooler subsurface water and strengthened equatorward transport, conducive to increased sea ice. As the majority of models underestimate summer jet trends, this cooling process is underestimated compared with observations and is insufficient to offset warming in the models. Through the sea ice-albedo feedback, models produce a high-latitude surface ocean warming and sea ice decline, contrasting the observed net cooling and sea ice increase. A realistic simulation of observed wind changes may be crucial for reproducing the recent observed sea ice increase.

## Abstract (web summary)

Mechanisms responsible for the recent increase in Antarctic sea ice cover remain elusive. Here, the authors show that model underestimates of changes in wind-induced ocean circulation may contribute, in part, to the failure of CMIP5 models to accurately capture modern Antarctic sea ice trends.

## Graphical abstract

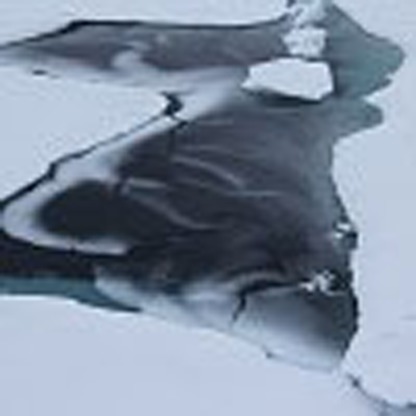

Despite regional melting, total Antarctic sea ice has been expanding over the past 35 years^1^,^2^,^3^. Such changes have an impact on surface albedo and deep water formation, and thus are important to global climate. Spatial analysis of sea ice concentration (SIC) trends reveals opposing regional changes since satellite observations began in 1979; decreasing sea ice in the Amundsen and Bellingshausen Seas is outweighed by increasing sea ice in the Ross Sea and around eastern Antarctica, leading to an overall increase^4^,^5^,^6^ (Fig. 1a). Although the circumpolar ice increase is statistically significant, it may still be within the range of natural variability^6^,^7^,^8^,^9^,^10^,^11^. However, as the most recent years of the sea ice record are included, the strength and statistical significance of the trend has increased^12^.

The sea ice increase has been attributed to regional-scale wind trends causing both dynamic and thermodynamic changes^5^,^10^,^13^,^14^,^15^,^16^. On the other hand, models have linked hemispheric-scale wind changes associated with the positive trend in the Southern Annular Mode^17^ (SAM), attributed to increasing greenhouse gases and stratospheric ozone depletion^18^,^19^,^20^, to Southern Ocean warming and a sea ice decline^21^,^22^,^23^. This contrasts interannual variations, in which a positive SAM intensifies the westerly jet and shifts it polewards, resulting in cool sea surface temperature (SST) and increased sea ice extent (SIE) at most longitudes due to enhanced Ekman drift^24^,^25^,^26^. An exception is along the Antarctic Peninsula, where a positive SAM is associated with reduced sea ice, due to circulation changes associated with the Amundsen Sea low^4^,^27^.

Most Coupled Model Intercomparison Project phase 5 (CMIP5) models fail to simulate the observed SIE increase in their historical experiments^7^,^8^,^9^,^11^,^28^,^29^. The vast majority of models produce a decrease in SIE and simulate considerable bias in mean-state SIE and its seasonal cycle^28^. The observed increase is suggested to lie within the range of modelled natural variability^7^,^8^,^29^, although modelled Antarctic sea ice variability tends to be overestimated^9^,^28^; in addition, when the spatial pattern of sea ice trends are considered, the observed changes are distinguishable from the modelled pattern during austral summer and autumn^30^. To date, few studies have proposed physical mechanisms that may be responsible for the difference between observed and simulated Antarctic sea ice trends^29^,^31^,^32^.

This study compares physical mechanisms affecting Antarctic sea ice in the CMIP5 models and observations, with the aim of explaining the difference between the observed increase and the modelled decline. We analyse monthly mean observations and output from 41 CMIP5 models with 87 realizations, over 1979–2013, the period for which regular satellite observations are available. Observed and modelled trends are assessed, and inter-model relationships used to gain insight into why models overall generate too great a sea ice loss and what the important processes behind this are. As the CMIP5 models underestimate recent changes in the SAM and the westerly wind jet intensification^7^,^33^,^34^,^35^, we investigate the influence of jet trends on Antarctic sea ice and Southern Ocean SST. We find that underestimated changes in wind-induced ocean circulation in the models may contribute, in part, to their large Antarctic sea ice decline.

## Results

### Sea ice and SST trends

**Figure 1:**
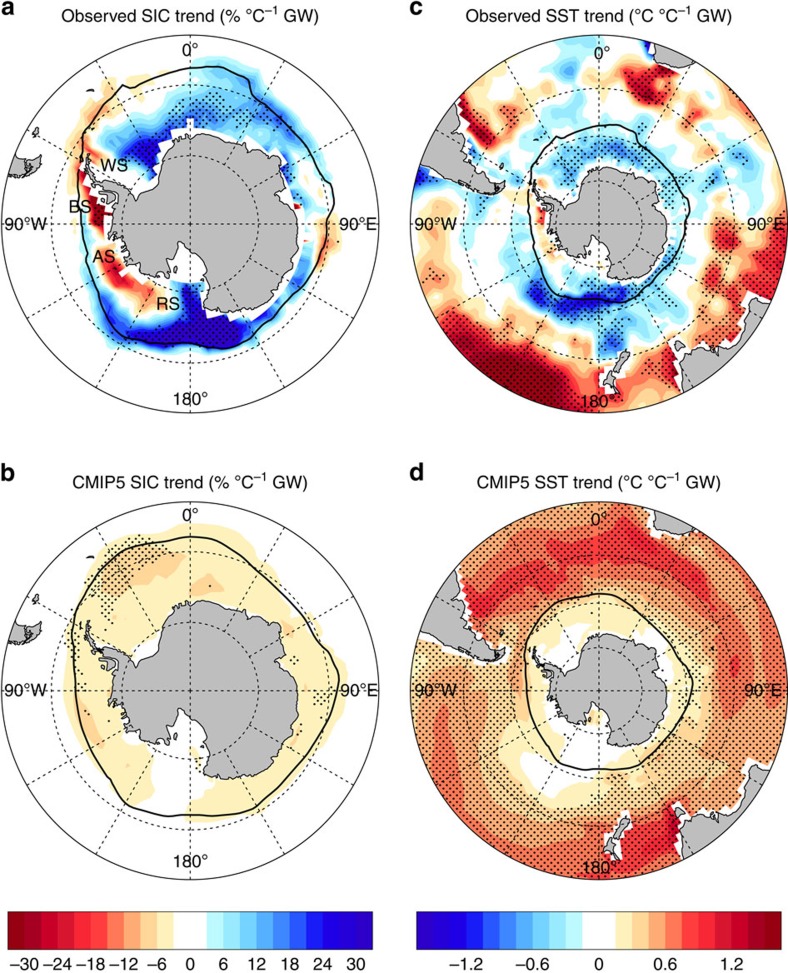
Annual SIC and SST trends over 1979–2013. (**a**) Observed SIC from the National Snow and Ice Data Center (NSIDC) Bootstrap algorithm, (**b**) CMIP5 multi-model mean SIC, (**c**) observed SST from Hadley Centre Sea Ice and Sea Surface Temperature (HadISST) and (**d**) CMIP5 multi-model mean SST. Trends are expressed as a change per degree of global warming (°C^−1^ GW). Multi-model means are calculated using the first available ensemble member for each model. Stippling indicates significance: (**a**,**c**) above the 95% level as determined by a two-sided Student's *t*-test and (**b**,**d**) where 80% of models agree on the sign of the mean trend^33^, which corresponds to 33 out of 41 models. The mean-state 15% SIC contour is shown in black. In (**a**) AS, Amundsen Sea; BS, Bellingshausen Sea; RS, Ross Sea; WS,Weddell Sea.

In contrast to the observed (Fig. 1a), multi-model mean SIC trends (Fig. 1b) show a decrease in all sea ice regions. The majority of models show an overall decrease in sea ice, despite inter-model variations in simulated spatial patterns, with many models showing small regions of increasing SIC (Supplementary Fig. 1). The multi-model mean regional decrease lacks broad coherence, except in the Bellingshausen and northern Weddell Seas, and in isolated pockets of eastern Antarctica and the Ross Sea. Coincident with the observed increase in Antarctic sea ice, high-latitude SST has also decreased over 1979–2013 (Fig. 1c)^10^,^31^,^36^, with cooling strongest in the Ross Sea. In contrast, the CMIP5 models show Southern Ocean surface warming over most regions (Fig. 1d), although there is no inter-model consensus in terms of warming at high latitudes.

**Figure 2:**
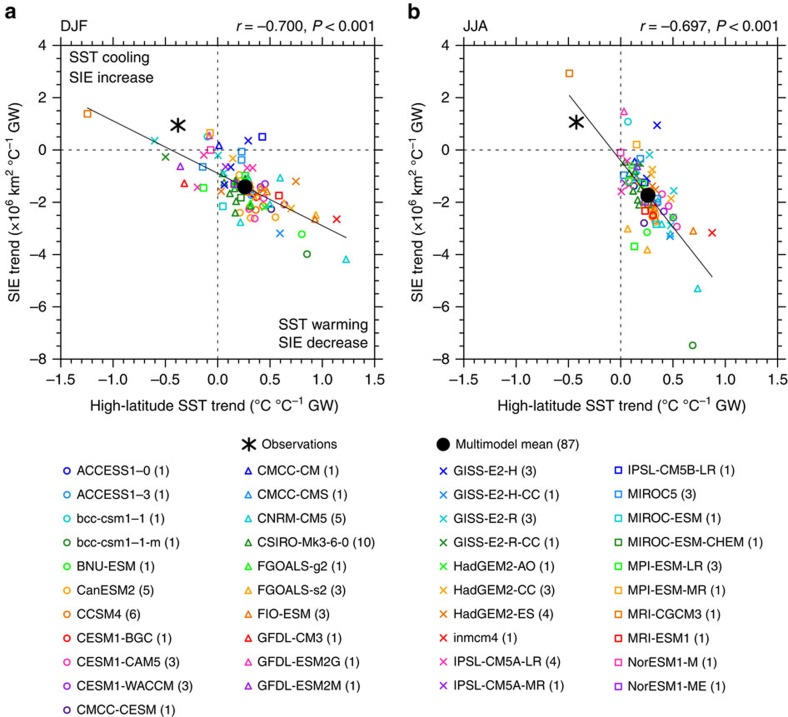
Trends in SIE versus trends in high-latitude SST over 1979–2013. (**a**) DJF and (**b**) JJA. Trends are expressed as a change per degree of global warming. All available model ensemble members are shown (87 realizations). Observed SST from HadISST and SIE from NSIDC. Each model is shown by a marker with the number of runs per model indicated in the legend, the multi-model mean is shown by a black dot and observations are shown by a black asterisk. The inter-model correlation coefficient and *P*-value are shown above each panel. For *P*<0.05, the inter-model regression is shown by a black line.

Comparing SIC and SST trend patterns in individual models (Supplementary Figs 1 and 2) reveals that models with stronger SST warming show a larger SIC decrease, as expected^7^. A strong inter-model relationship exists between trends in area-averaged high-latitude (south of 55°S) SST and in circumpolar SIE: models that simulate greater warming produce a greater reduction in ice (Fig. 2). This relationship is highly statistically significant (*P*<0.001) and is evident in all seasons (shown for summer (December–February (DJF)) and winter (June–August (JJA); Fig. 2a,b, respectively; all seasons in Supplementary Table 1). The observed trends fit the tail-end of the spread in model trends. When trends are scaled by global mean temperature trends to take into account differences in climate sensitivity between observations and models and between models (Fig. 2), the observed SIE trend lies outside the 95% confidence interval of model trends. This is despite that in absolute terms the observed Antarctic sea ice trend is not statistically distinguishable from the modelled trends at the 95% confidence level. As such, natural internal variability remains a possible contributing factor for the observed trend. However, it is still of interest to investigate mechanisms that lead to the range in observed and modelled SIE trends. The SIE–SST relationship (Fig. 2) suggests that ocean changes may influence sea ice trends. As such, to explain why the majority of CMIP5 models simulate a decrease in Antarctic sea ice in contrast to the observed increase, we must understand why modelled high-latitude SST warms too fast.

### Sea ice and SST relationships with the westerly jet

**Figure 3:**
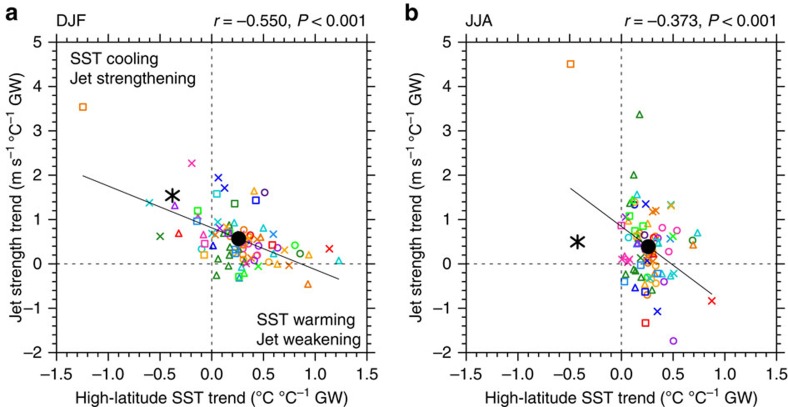
Trends in jet strength versus trends in high-latitude SST over 1979–2013. (**a**) DJF and (**b**) JJA. Trends are expressed as a change per degree of global warming. All available model ensemble members are shown. Observed jet strength from European Centre for Medium-Range Weather Forecasts Interim Reanalysis (ERA-Interim). Figure details as per Fig. 2.

Given the links between the westerly wind jet, sea ice and SST^21^,^22^,^23^,^24^,^25^,^26^, we next investigate the influence of jet intensification trends. There is a significant inter-model relationship between jet strength trends and SIE trends during summer and autumn (*P*<0.01; Supplementary Table 1). During these seasons, there is also a strong and significant relationship between high-latitude SST and jet strength (*P*<0.001; Fig. 3a and Supplementary Table 1). In contrast, during winter there is no inter-model relationship between jet strength trends and SIE trends, although the relationship between jet strength trends and SST trends persists (*P*<0.001; Fig. 3b). This relationship shows that models with a more intensified jet cool, or warm less, whereas models with a weaker intensification, or weakened jet, warm more. In summer, significant relationships are also found between trends in jet position and high-latitude SST, with a stronger poleward shift in the jet associated with high-latitude SST cooling or weaker warming (Supplementary Table 1).

Processes embedded in the inter-model trend relationships appear to also operate in the inter-model relationship in the mean state: models with stronger mean-state zonal winds south of 55°S tend to have a larger ice area, in particular during autumn^7^. On interannual timescales, an intensified summer and autumn jet is associated with above-average SIE in the observations (*P*<0.05; Supplementary Table 2 and Supplementary Fig. 3). However, the majority of CMIP5 models do not capture this interannual relationship (median *P*>0.2), indicating a failure to simulate wind–ice interactions adequately. This may explain the somewhat weak inter-model relationship between trends in jet strength and SIE (Supplementary Table 1).

Within individual CMIP5 models, interannual jet strength is more strongly correlated with high-latitude SST (Supplementary Table 2 and Supplementary Fig. 3). An intensified jet is associated with cooler high-latitude SST^24^,^25^. The observed relationship is significant in summer (*P*<0.05), whereas for the majority of CMIP5 models it is statistically significant in both spring and summer (median *P*<0.05 and *P*<0.01, respectively). Thus, relative to observations, variations in the modelled jet have a weaker influence on variations in sea ice, yet more influence on SST. As such, we focus on jet–SST dynamics, noting the strong relationship between SST and SIE (Fig. 2) that links wind changes back to sea ice.

### Mechanism for wind-induced effects on SST

We hypothesize that the jet–SST trend relationship in the CMIP5 models is conducted through a high-latitude Ekman response to changing winds. There, the wind stress forces equatorward Ekman transport and the wind stress curl forces upward Ekman pumping^36^,^37^ (see Methods). As such, an intensified jet results in strengthened equatorward Ekman transport and usually increased Ekman upwelling at high latitudes.

**Figure 4:**
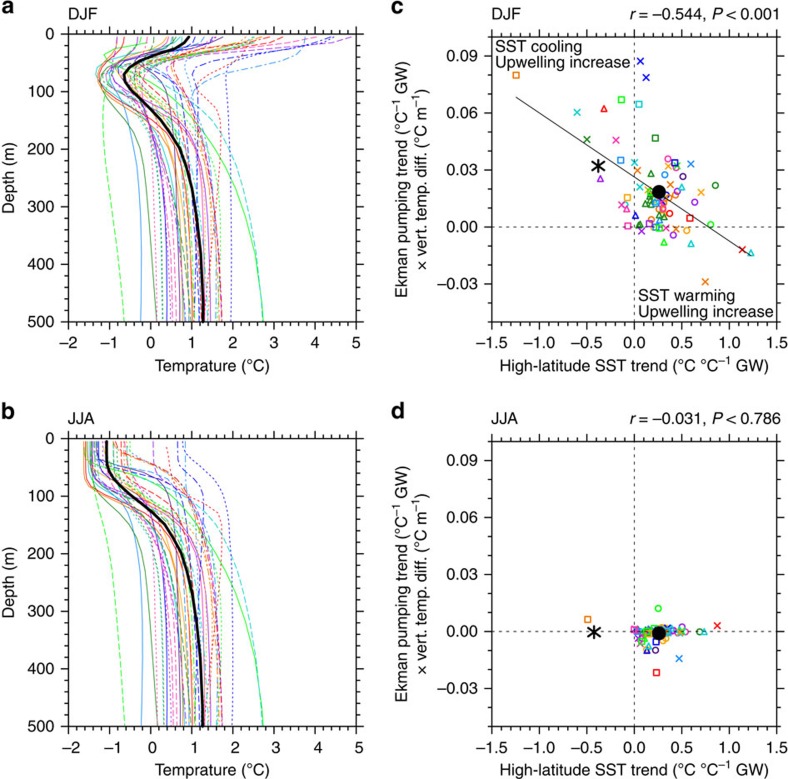
Potential temperature profiles and Ekman pumping trends over 1979–2013. Seasonal zonal–mean potential temperature profiles averaged over 60–70°S for (**a**) DJF and (**b**) JJA. The first available ensemble member for each model is shown. The observed profile (black) is an average of Simple Ocean Data Assimilation (SODA) and Ishii reanalyses over 1979–2011. Trends in Ekman pumping versus trends in high-latitude SST for (**c**) DJF and (**d**) JJA. Trends are expressed as a change per degree of global warming. Ekman pumping trends are calculated as the trend in the Ekman pumping velocity principal component (PC) multiplied by the mean-state vertical temperature gradient near the surface. All available model ensemble members are shown. Details in (**c**,**d**) as per Fig. 2.

Ekman upwelling has a strong cooling effect on SST during summer when warm water resides at the surface forming a cap over cool Winter Water at depths ∼20–150 m (Fig. 4a). The warm surface water results from short-wave radiation being received by the summer ice-free surface waters as sea ice melts. Beneath this, the permanent pycnocline with cold, fresh water overlying warm, salty water is apparent. By contrast, during winter surface waters are colder than water below (Fig. 4b), consistent with the typical temperature profile described for the high-latitude Southern Ocean^37^, caused by seasonal sea ice melt/freeze and advection processes that freshen the surface layer. Because of the seasonal stratification, during summer enhanced Ekman upwelling brings cooler waters to the surface and this surface cooling spreads further north due to enhanced equatorward transport.

Consistently, summer Ekman pumping trends are significantly correlated with SST trends at high latitudes (*P*<0.001; Fig. 4c): models with a strong increase in Ekman upwelling show SST cooling or weak warming, whereas models with weak trends in Ekman pumping show strong SST warming. Ekman transport trends are also significantly correlated with high-latitude SST trends in summer (*P*<0.001; Supplementary Fig. 4): models with a strong increase in equatorward Ekman transport show cooling or weak warming. Scale analysis^38^ has suggested that horizontal Ekman transport should initially dominate over vertical Ekman upwelling, although here we find that south of ∼60°S, both Ekman transport and Ekman pumping are important (see Methods and Supplementary Fig. 5).

The CMIP5 models underestimate the summer intensification (Fig. 3a) and poleward shift in the jet^7^,^34^, and therefore also underestimate the increased upward Ekman pumping (Fig. 4c) and equatorward Ekman transport (Supplementary Fig. 4c) compared with observed trends. Many models underestimate the vertical temperature advection despite overestimating the surface stratification during summer (Fig. 4a). This contributes to their high-latitude SST warming trends in contrast to observed cooling.

There is considerable uncertainty in the observed jet trend, owing to sparse observations over the high-latitude Southern Hemisphere^19^,^35^. Although only ERA-Interim jet trends are presented here, stronger jet intensification is also seen in three other reanalyses (see Methods^39^). Increased wind speed is also evident in station-based wind observations^40^,^41^. However, satellite-based wind observations available over the shorter 1988–2011 period may cast some doubt over reanalysis trends^39^, although the satellite products themselves contain uncertainty^42^. Overall, the analyses presented here depend on an accurate wind trend estimate over the Southern Ocean, however the mechanisms described remain robust. Further, although the observed (ERA-Interim) jet intensification is stronger than the multi-model mean, it does lie within the model spread (Fig. 3a). Nevertheless, as discussed above, the strong inter-model relationship shown here suggests that the strength of jet intensification is an important process influencing high-latitude SST in observations and coupled models, and the majority of models produce a weaker than observed intensification.

No significant relationship between trends in Ekman pumping and SST exists during winter (Fig. 4d and Supplementary Table 1). This is to be expected, given the seasonal variation in vertical temperature stratification: during winter, increased upwelling would cause warming, offsetting cooling from increased equatorward transport (Supplementary Table 1).

### Timescales of Ekman response

On interannual timescales, a positive SAM is associated with cool high-latitude SST^24^,^25^,^26^, whereas over longer periods a positive trend in the SAM has been linked to high-latitude SST warming^21^,^22^,^23^. The apparent contradiction between the SAM–SST relationship over inter-annual versus multidecadal timescales has been explained by a two-timescale SST response to high-latitude wind changes^36^,^38^. Initially, a positive SAM trend is associated with short-term cooling, by increasing the northward Ekman transport of cold surface waters in the prevailing westerly wind regions^25^,^36^,^38^, consistent with the inter-model trend relationship here (Supplementary Fig. 4). Over time, however, the cooling is replaced with a warming, accounted for by prolonged enhanced upwelling in a region where a temperature inversion occurs^21^,^22^,^23^. Our results are consistent with these previous studies in that most CMIP5 models simulate a positive SAM trend (that is, jet intensification; Fig. 3a), increased upwelling and high-latitude SST warming (upper right quadrant of Fig. 4c). Our results suggest, however, that over the 35-year period assessed, Ekman upwelling is not responsible for the surface warming during summer, because due to the seasonal stratification profile models that simulate a stronger upwelling trend show a weaker rate of surface warming. Instead, the inter-model relationship suggests that cooling due to Ekman upwelling offsets other warming factors. Importantly, most models underestimate the increase in Ekman upwelling, resulting in a weaker cooling effect that is insufficient to offset warming from other processes, most notably surface heat fluxes.

The seasonal variation in the subsurface temperature profile is not discussed in previous studies and is key to interpreting our results: in contrast to previous studies that link initial cooling to equatorward Ekman transport only^36^,^38^, our results suggest that Ekman pumping during summer is also important. It is plausible that the cooling associated with summer upwelling may eventually be replaced by warming, as water from below the mixed layer is entrained^36^,^38^; however, over the timescale assessed here (1979–2013), this does not appear to be the case. In previous model experiments, the time required for this temperature-trend transition varies from an order of years to a couple of decades^38^. If the timescale to a complete transition from initial cooling to later warming was at the longer end of this estimate, then the surface cooling seen in the observations and in some models could be consistent with this mechanism. The seasonal variation in the temperature profile may also contribute to a longer transitional timescale.

### Ekman contribution to observed modelled disparity

The mechanism identified above is present during summer. We estimate that >25% of the difference in the CMIP5 SIE trends can be attributed to their underestimated jet intensification (see Methods). As such, the underestimation of westerly wind trends in CMIP5 models probably contributes to the sea ice decrease simulated by the majority of models.

Owing to the thermal inertia of the ocean, SST anomalies in summer are likely to persist and exert an influence beyond this season. Observed spring ice tendencies have been found to persist until the following winter^43^,^44^, and here we find that in both observations and models summer ice tendencies persist significantly during autumn and winter. This confirms that the summer Ekman–SST mechanism can influence trends throughout much of the year^23^,^38^.

Positive feedbacks associated with the wind-induced circulation changes could also contribute to the difference between observed and modelled trends. In observations, the magnitude of Ekman pumping and transport increases such that the associated cooling more than offsets the high-latitude heat flux increase. Cooler SST leads to increased sea ice, in particular in the Ross Sea sector. Through the sea ice-albedo feedback, solar radiation decreases, conducive to further cooling and increased sea ice^43^. The consequence is decreased zonal-mean downward heat flux in the sea-ice zone (Supplementary Fig. 6c). In the majority of the models, although the magnitude of Ekman pumping and transport also increases, it is to a smaller extent such that the associated cooling is not sufficient to offset the heat flux increase. The above sea ice-albedo feedback process operates in reverse, leading to an increased heat flux into the ocean in the sea-ice zone (Supplementary Fig. 6d). Based on the occurrence of opposite reinforcing feedback mechanisms occurring in observations and the majority of CMIP5 models, the difference between observed and modelled jet strength trends can lead to very different sea ice changes.

### Considering other mechanisms

Further support for the importance of Ekman upwelling and transport comes from considering other potential mechanisms. Here we explore other possible processes and find that none of these contradict or offer an alternative explanation for the results described above.

It could be hypothesized that models with stronger jet trends show reduced high-latitude SST warming as a result of changed cloud cover or evaporative cooling, and that the correlations presented above between Ekman pumping and SST (Fig. 4c) are coincidental. No inter-model relationship is found between trends in SST and trends in overlying cloud cover during summer (*P*>0.15; Supplementary Table 3), possibly due to differing cloud–jet relationships present in the models^45^. A significant inter-model relationship is found between high-latitude SST trends and evaporation trends (*P*<0.001; Supplementary Table 3), in which models with increasing SST show an increase in evaporation and vice-versa. This suggests that SST anomalies are driving evaporation variations, as warmer waters evaporate more readily, whereas evaporative cooling would have the opposite effect on SST. Thus, evaporative fluxes are not the cause of the excessive warming in most CMIP5 models, instead they are a response to this warming. These results support our hypothesis above; namely, that the relationship between the westerly wind jet and SST trends occurs due to changes in ocean circulation.

Outside of the summer sea-ice zone, spatial trends in total downward heat flux oppose those in SST (Supplementary Fig. 6), that is, regions of SST cooling are associated with increased heat flux into the ocean, suggesting that changes in SST are driving changes in heat flux and not the other way around. During summer, the inter-model relationship between trends in SST and surface heat fluxes over these predominantly ice-free areas (55–65°S) is insignificant, although the sense of the relationship suggests that models with SST cooling show increased heat flux into the ocean (Supplementary Fig. 7), again suggesting that changes in SST are driving changes in heat flux. Thus, net heat flux trends are of the wrong sign to account for model SST trends. It is also noted that area-averaged trends in observed heat fluxes, although uncertain, are comparable to those in CMIP5 models over this region.

Southern Ocean freshening due to accelerated Antarctic ice shelf and/or ice sheet melting, not simulated by CMIP5 models, was proposed as a possible mechanism contributing to the Antarctic sea ice increase^31^. However, further model experiments found the influence of ice sheet melt on sea-ice trends to be minimal^29^. As such, this deficiency alone in CMIP5 models cannot account for the disparity between observed and simulated Antarctic sea ice trends. Nevertheless, reduced Southern Ocean convection in CMIP5 models has been linked with surface freshening^46^, suggesting that overall changes in freshwater fluxes may be important for surface temperature trends^47^.

Inter-model trends in SIE are strongly related with trends in sea surface salinity (*P*<0.001; Supplementary Table 3 and Supplementary Fig. 8a): models with increasing salinity show a strong decrease in sea ice, whereas models with surface freshening show an increase, or weaker decrease, in sea ice. The sense of this relationship suggests that ocean surface salinity is influencing sea ice, not the other way around, as sea-ice-driven surface salinity changes would see freshening correspond to higher rates of sea-ice melt. Instead, greater sea-ice coverage is linked to fresher surface conditions and increased surface stratification, which suppresses convective overturning and vice versa for reduced sea-ice coverage.

Sea-surface salinity trends cannot be explained by trends in high-latitude precipitation minus evaporation (*P*−*E*), as surprisingly increasing *P*−*E* is associated with increasing salinity (*P*=0.05 during summer; Supplementary Table 3 and Supplementary Fig. 8b), opposite to what would be expected, because both *P*−*E* and surface salinity trends co-vary with the jet. Again, changes in wind-induced ocean circulation provide the probable explanation. Namely, surface salinity trends are weakly related with Ekman pumping trends (*P*<0.1 during summer; Supplementary Table 3 and Supplementary Fig. 8c): models with a stronger increase in Ekman upwelling show an increase in surface salinity, whereas models with a weaker increase (or decrease) in Ekman upwelling show a decrease in surface salinity, due to the upwelling (or lack thereof) of saltier water from depth (Supplementary Fig. 8d). The temperature response associated with the Ekman upwelling of salty subsurface water (warming due to decreased stability and increased convective overturning) dampens the direct Ekman–SST response (upwelling of cool Winter Water) and this may be the reason why no significant Ekman–SIE relationship is found directly in the models over the 35-year period assessed.

## Discussion

**Figure 5:**
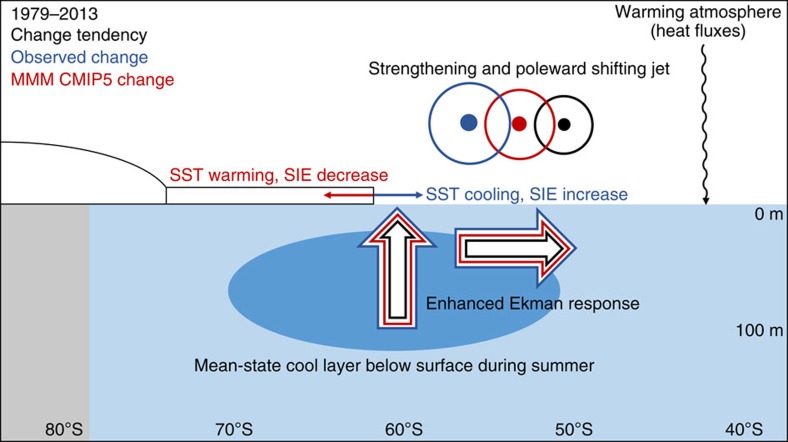
Schematic of decadal scale wind induced surface layer changes during summer. Over recent decades, the observed westerly wind jet has strengthened and shifted poleward during austral summer (circles with dots). This has increased the upward Ekman pumping and equatorward Ekman transport (large arrows). During summer, increased upwelling at high latitudes brings cooler Winter Water to the surface. Combined with equatorward transport, this leads to SST cooling in observations. Multi-model mean (MMM) CMIP5 changes (red) are weaker than observed changes (blue). Under global warming, these weaker Ekman changes are insufficient to offset warming from other factors (curvy arrow). As such, multi-model mean CMIP5 high-latitude SST has warmed rather than cooled and Antarctic sea ice has declined rather than expanded (small arrows).

Both the observed and CMIP5 SIE trends are linked to the westerly wind jet intensification through the influence of SST. The models underestimate the observed jet intensification during summer, although we caution that the observed jet trend is uncertain. This causes a weaker strengthening of high-latitude Ekman pumping and transport than observed. Although increased Ekman upwelling of cool Winter Water and the associated equatorward Ekman transport contribute to the observed SST cooling, because their trends are underestimated in the models, these terms are insufficient to offset warming from increased surface heat fluxes. This leads to faster surface warming and a decreasing sea-ice trend in most of the models (summarized in Fig. 5). Once these trends are initiated, the sea ice-albedo positive feedback ensures the trend is sustained. These findings demonstrate the importance of accurately simulating changes in the wind^7^,^48^. By contrast, in the observations, the cooling effect from the wind changes appears to be sufficient to offset the warming tendency resulting in an initial cooling. The same sea ice-albedo positive feedback operates in reverse, leading to further cooling and an increasing sea ice trend. Although analyses are largely conducted over circumpolar regions, when repeated for the Ross Sea sector, where observed SIE has increased most substantially, results remain robust.

Our finding that underestimated wind trends contribute to the discrepancy between the observed and model sea-ice changes occurs despite the fact these models do not resolve eddies, although almost all include a suitable eddy-induced advection scheme to approximate their effects. In the real world eddy compensation would partially counteract wind-induced changes^38^,^49^, although Ekman changes still dominate in the surface layer^50^,^51^. In the presence of an eddy compensation effect, the underestimation of the impact in the models could be even larger. This influence and others such as the role of deep ocean overturning or convection will provide fertile ground for further research into the recent Southern Ocean circulation and sea ice changes.

## Methods

### Data

CMIP5 data from the historical and Representative Concentration Pathway 8.5 (RCP8.5; high-emission scenario) experiments are concatenated to match the observational period. The choice of RCP scenario over 2006–2013 has minimal influence on results, as all forcing scenarios are very similar over this time frame. We analyse all CMIP5 models that have SIC data available for both the historical and RCP8.5 experiments. This includes 41 CMIP5 models, with a total of 87 realizations (between one and ten runs are available per model), listed in the legend of Fig. 2. We also make use of SST, potential temperature, sea surface salinity, subsurface salinity (historical experiment only), surface air temperature, zonal wind, surface wind stresses, evaporation, precipitation, total cloud cover, mean sea level pressure and surface heat fluxes from the CMIP5 archive. At the time of analysis, potential temperature was not available for Flexible Global Ocean-Atmosphere-Land System model spectral version 2 and First Institute of Oceanography Earth System Model, and salinity was not available for Hadley Centre Global Environment Model version 2 Atmosphere-Ocean. Various surface heat flux terms were not available for Centro Euro-Mediterraneo sui Cambiamenti Climatici Climate Model with a resolved Stratosphere, First Institute of Oceanography Earth System Model, Goddard Institute for Space Studies ModelE/Russell (r1i1p2 only), Hadley Centre Global Environment Model version 2 Atmosphere-Ocean, Max Planck Institute Earth System Model Low Resolution (r2i1p1 and r3i1p1 only) and Meteorological Research Institute Earth System Model version 1.

For comparison with observations, we use passive microwave SIC processed using the NSIDC Bootstrap algorithm^2^. The possibility for spurious trends in this SIC data set has been identified^52^; thus, the results are compared with those obtained with SIC processed using the National Aeronautics and Space Administration Team algorithm and are found to be very similar. For area-averaged SIE, we make use of the pre-calculated NSIDC SIE index^53^, as this index is commonly used in other studies^11^,^12^. Results calculated using the NSIDC Bootstrap SIC are very similar. We use SST data from the HadISST data set^54^. For ocean temperature and salinity, we take an average of the SODA v2.2.4 (ref. 55) and Ishii^56^ reanalyses. For atmospheric variables, we use the ERA-Interim reanalysis^57^, regarded as the most reliable reanalysis over the Amundsen and Bellingshausen Seas^34^, and over Antarctica^58^,^59^. Uncertainty exists in ERA-Interim wind trends^39^; however, considering the trends evident in the National Centers for Environmental Prediction (NCEP)/National Center for Atmospheric Research reanalysis, NCEP/Department of Energy reanalysis and Twentieth Century reanalysis v2, ERA-Interim winds may modestly underestimate the jet intensification, as this product yields the weakest trend among these four reanalysis products^39^. Weaker jet intensification is seen in National Aeronautics and Space Administration Modern Era-Retrospective analysis for Research and Applications and NCEP Climate Forecast System Reanalysis, although these products have previously been excluded when examining Southern Ocean wind strength trends, due to possible issues with reanalysis data assimilation^35^. The overall balance of evidence suggests that ERA-Interim winds provide one of the best estimates of wind trends over the Southern Ocean for the full study period of 1979–2013.

All data are bilinearly interpolated to a standard 2° × 2° grid. This resolution is chosen to avoid overextrapolating low-resolution data from some models to higher resolutions. Potential temperature is converted from *σ* to *z*-levels where required and vertically interpolated to 40-depth levels (matching the SODA reanalysis). Data are stratified into seasonal and annual mean fields. The year of an austral summer corresponds to the year of the January–February.

### Metrics

A number of metrics are calculated for observations and each model. Time series of metrics are used to investigate and compare linear trends and interannual variability between models and observations. For inter-model relationships, each ensemble member is included in the analysis and weighted evenly. Linear trends are calculated using the least squares regression method and are scaled by linear trends in global-mean temperature to take into account the different climate sensitivity of the models. Statistical significance is determined using the two-sided Student's *t*-test. When assessing the significance of interannual correlation coefficients, the lag-1 autocorrelation is accounted for by estimating the effective sample size, *N*_eff_, as:


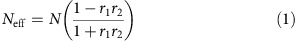


where *N* is the sample size, and *r*_1_ and *r*_2_ are the lag-1 autocorrelations of the time series of interest^60^.

SIE in the models is defined as the circumpolar area where SIC exceeds 15% (ref. 28). We focus on SIE as an area-averaged metric, as it is commonly assessed^7^,^8^,^9^,^28^,^29^, although we also present SIC trends to display regional trend characteristics (Fig. 1). High-latitude metrics (for example, SST, sea surface salinity and *P*−*E*) are defined as the area-averaged field south of 55°S (except where noted otherwise). The choice of latitude is assessed and results are found to be robust over a range of high latitudes. Only ocean grid points are considered in area averages. In HadISST, SST in grid cells partially covered by sea ice is determined based on a statistical relationship between SST and SIC^54^. In the CMIP5 models, SST is defined as the temperature of the uppermost model layer. Jet strength is defined as the maximum 925-hPa westerly wind between 35–70°S, where a cubic spline approximation is applied to the zonal–mean zonal wind^34^.

Meridional Ekman transport, *V*_E_, is calculated from the surface zonal wind stress 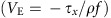 and Ekman pumping, *w*_E_, from the curl of surface wind stresses 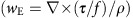, where **τ** is the wind stress, *ρ* is the density of seawater and *f* is the Coriolis parameter. Trends calculated from area-averaged Ekman transport and pumping time series are sensitive to the choice of latitude band, owing to variations in the wind fields among models and observations. To allow for spatial variations among models, we calculate the first empirical orthogonal function (EOF) of both Ekman transport and pumping over 55–70°S and use the standardized PCs to represent the Ekman transport and pumping time series, respectively. During summer, the first EOFs for both Ekman transport and pumping are well separated^61^ from subsequent patterns in all models and observations. The first EOFs are related to the SAM, the leading mode of atmospheric variability in the extratropical Southern Hemisphere^62^, and have a more coherent influence on SST (Supplementary Fig. 9). To account for the effect that equatorward Ekman transport has on SST, we estimate horizontal temperature advection by multiplying the Ekman transport PC by the mean-state horizontal temperature difference between 55–60°S, and 65–70°S, calculated for the zonal–mean surface layer (0–25 m). Likewise, to account for the effect that Ekman pumping has on SST, we estimate the vertical temperature advection by multiplying the Ekman pumping PC by the mean-state vertical temperature difference between the surface layer (0–25 m) and the layer just below the summer thermocline (70–80 m), calculated for the zonal–mean over 55–70°S.

### Scale analysis

Horizontal temperature advection due to Ekman transport is compared with vertical temperature advection due to Ekman upwelling (Supplementary Fig. 5). We use area-averaged terms for calculations so that various latitude bands can be assessed; as we are interested in mean-state orders of magnitude rather than linear trends, comparing area averages rather than PCs is reasonable.

Horizontal and vertical advection terms are compared as follows:


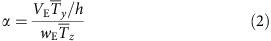


where 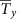 is the horizontal temperature gradient over the latitude bands assessed, *h* is the depth of the surface layer (25 m) and 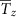 is the vertical temperature gradient at the base of the summer mixed layer. The inclusion of *h* in the numerator is necessary to directly compare terms as Ekman transport is a depth-integrated flow in the upper *h* metres, whereas Ekman pumping is a velocity.

Over all latitude bands assessed *V*_E_>>*w*_E_; however, 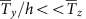. As a result, we find the numerator and denominator in equation (2) to be of similar orders of magnitude. Further equatorward, where *τ*_*x*_ is relatively large and ∇ × **τ** small, northward Ekman transport is larger and *α*∼5–10, but between 60–75°S, where the zonal wind transitions from westerly to easterly, *α*∼0.5–2. As such, we conclude that over these latitude bands, both Ekman transport and Ekman pumping terms are important in driving variations in SST, and hence sea ice.

### Estimating the Ekman contribution to modelled disparity

We use the difference between the observed (ERA-Interim) and multi-model mean jet strength trends and the sensitivity of SST trends to jet trends (that is, the inverse of Fig. 3a), to estimate the Ekman contribution to SST trends. From this, we use the sensitivity of SIE to SST (Fig. 2a), to further estimate the Ekman contribution to SIE trends.

## Additional information

**How to cite this article:** Purich, A. *et al*. Evidence for link between modelled trends in Antarctic sea ice and underestimated westerly wind changes. *Nat. Commun.* 7:10409 doi: 10.1038/ncomms10409 (2016).

## Supplementary Material

Supplementary Information Supplementary Figures 1-9 and Supplementary Tables 1-3.
