# Supplementary material for: Evidence for link between modelled trends in Antarctic sea ice and underestimated westerly wind changes
Source: Nat Commun. 2016 Feb 4;7:10409. doi: 10.1038/ncomms10409 (PMC4742833; doi:10.1038/ncomms10409)
Supplement: Supplementary Information — Supplementary Figures 1-9 and Supplementary Tables 1-3. [file ncomms10409-s1.pdf]

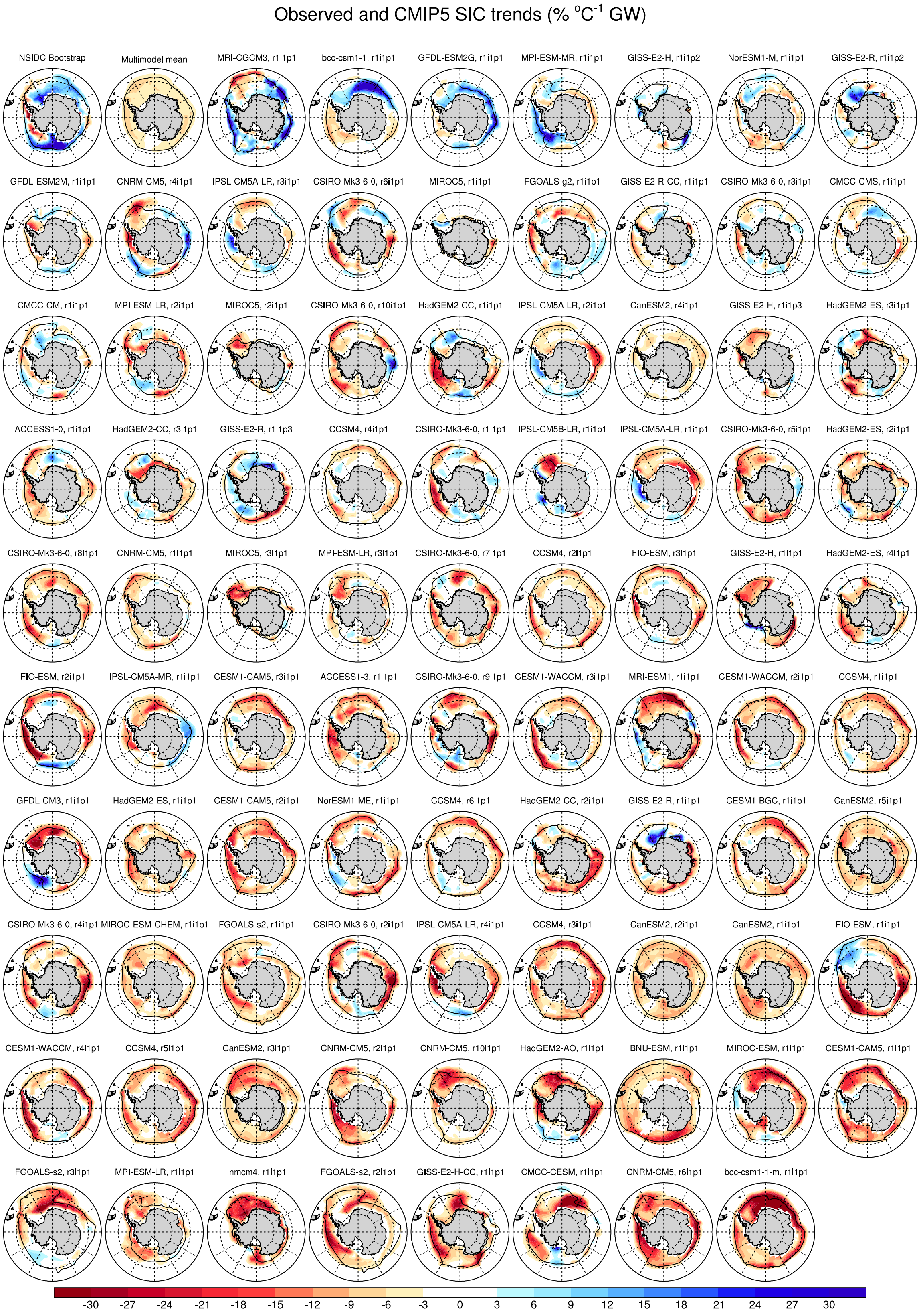

Supplementary Figure 1 | Annual SIC trends for observations and models over 1979–2013. Trends are expressed as a change per degree of global warming. (top left) Observed SIC from the NSIDC Bootstrap algorithm. (top second left) Multi-model mean. Individual models are ordered based on their annual SIE trend, with models with the largest SIE increase shown first (top rows) and models with the largest SIE decrease shown last (bottom rows).

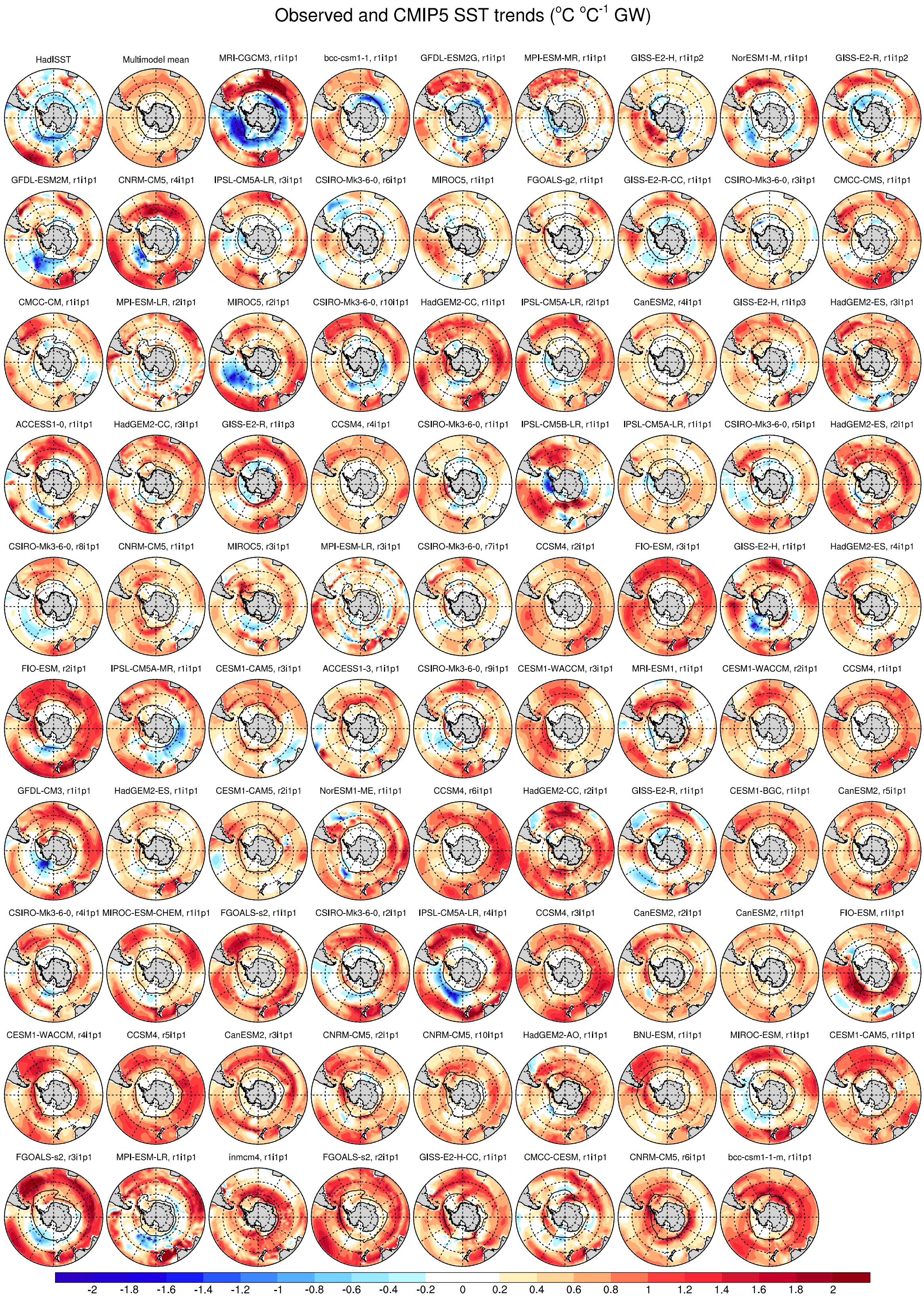

Supplementary Figure 2 | Annual SST trends for observations and models over 1979–2013. Trends are expressed as a change per degree of global warming. (top left) Observed SST from HadISST. (top second left) Multi-model mean. Individual models are ordered as per Supplementary Fig. 1.

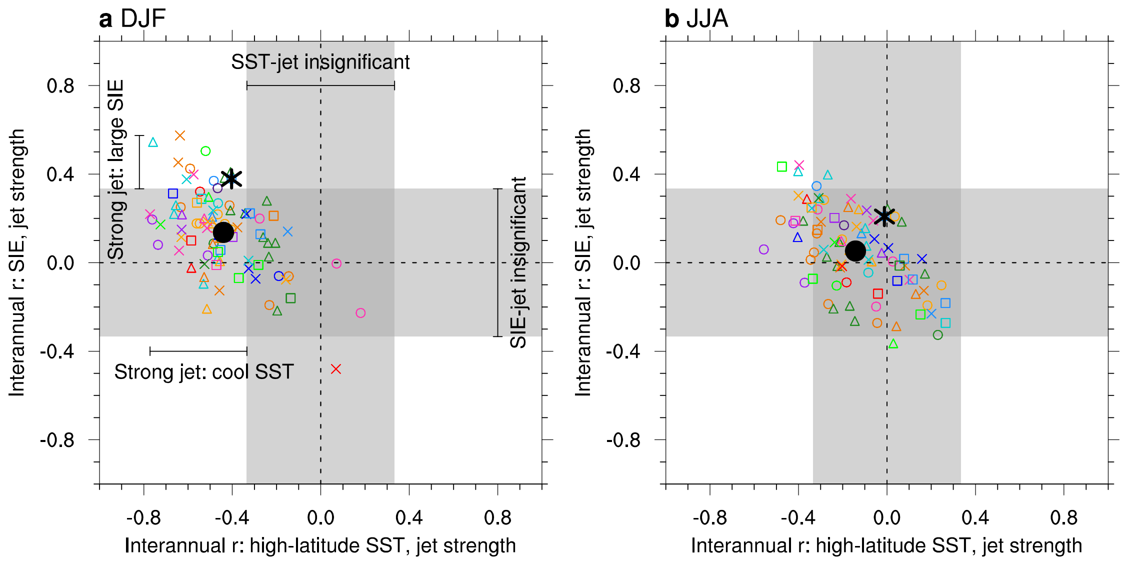

Supplementary Figure 3 | Interannual correlation coefficients between jet strength, high-latitude SST and SIE over 1979–2013. a DJF, and **b** JJA. Interannual correlations are calculated for observations and each model realisation separately, using linearly de-trended time series. The interannual correlations between high-latitude SST and jet strength are shown on the horizontal axes. The interannual correlations between SIE and jet strength are shown on the vertical axes. All available model ensemble members are shown. Observed SST from HadISST, SIE from NSIDC and jet strength from ERA-Interim. Figure details as per Fig. 2 of the manuscript. Grey shading indicates correlations insignificant at the 95% confidence level, as determined by a two-sided Student's *t* test. Autocorrelation is not accounted for in this shading, but has very little influence on the significance of individual models (p-values change by <0.005). SST-jet correlations within the vertical grey band are insignificant. SIE-jet correlations within the horizontal grey band are insignificant. Both SST-jet and SIE-jet correlations within the centre darker grey square are insignificant. Most of the JJA correlations shown in **b** lie in the centre darker grey square and are insignificant. In DJF (**a**), more correlations lie outside the vertical band of grey shading, but not outside the horizontal band of grey shading, indicating that in most models there is a stronger interannual jet-SST relationship then there is interannual jet-SIE relationship.

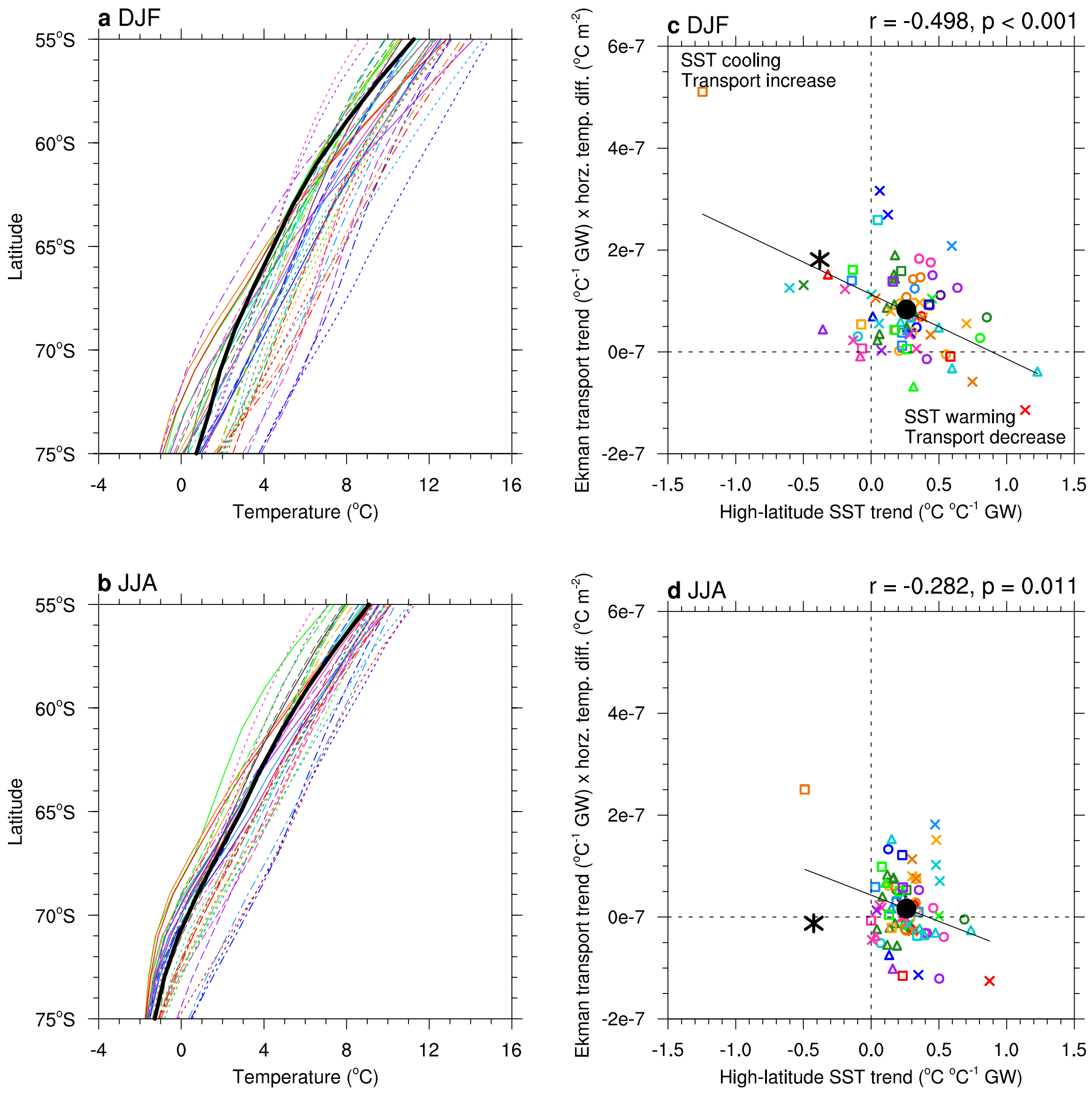

Supplementary Figure 4 | Zonal-mean temperature versus latitude, and Ekman transport trends over 1979–2013. Seasonal zonal-mean temperature averaged over 0–25 m for **a** DJF, and **b** JJA. The first available ensemble member for each model is shown. The observed profile (black) is an average of SODA and Ishii over 1979–2011. Trends in Ekman transport versus trends in high-latitude SST for **c** DJF, and **d** JJA. Trends are expressed as a change per degree of global warming. Ekman transport trends are calculated as the trend in the Ekman transport PC multiplied by the mean-state horizontal temperature gradient at high latitudes. All available model ensemble members are shown. Figure details as per Fig. 2 of the manuscript.

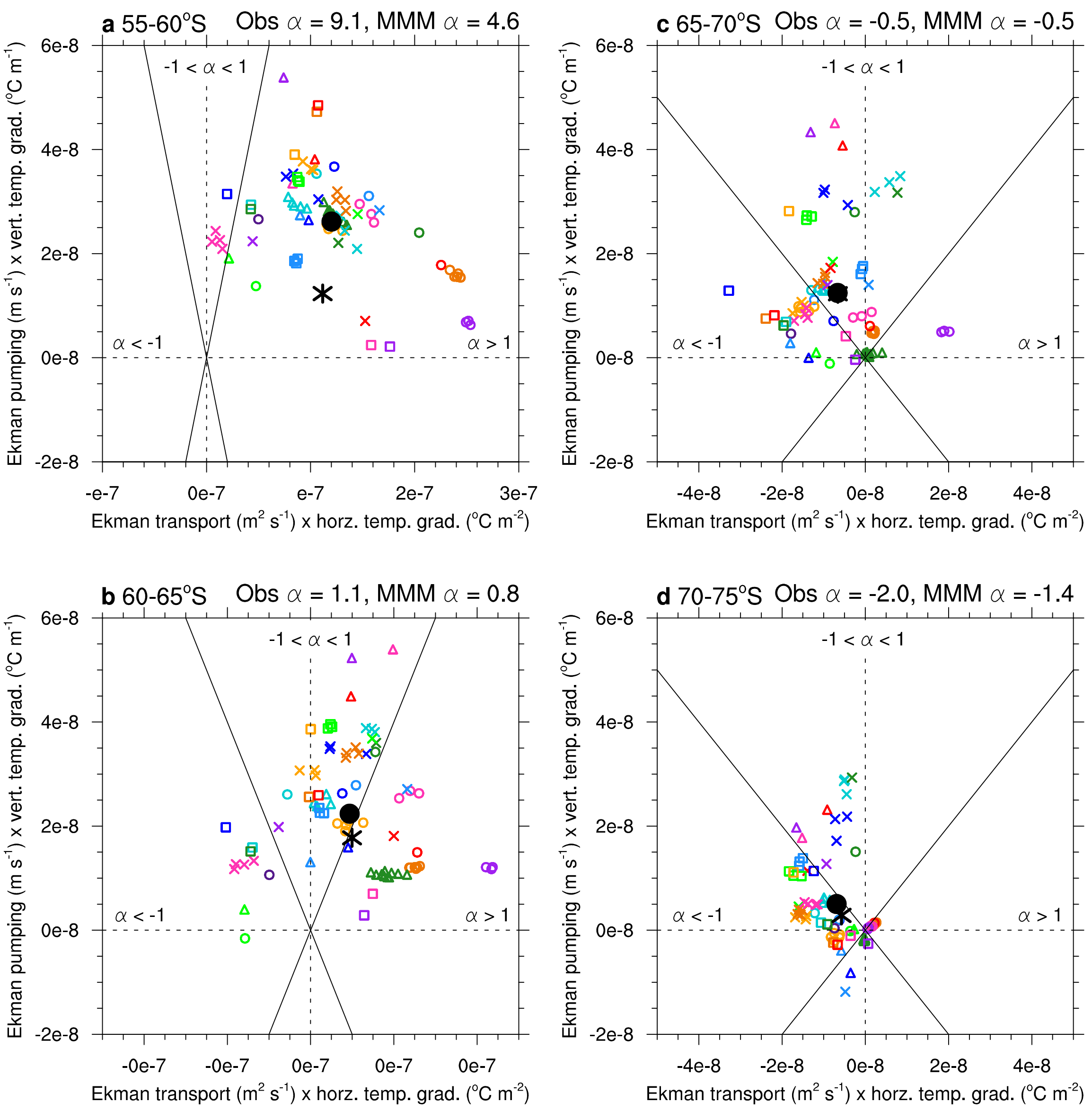

Supplementary Figure 5 | DJF horizontal versus vertical mean-state temperature advection over 1979–2013. Advection terms are averaged over **a** 55–60°S, **b** 60–65°S, **c** 65–70°S, and **d** 70–75°S. The horizontal axes show the numerator from Equation 2 of the manuscript (horizontal temperature advection) and the vertical axes show the denominator from Equation 2 of the manuscript (vertical temperature advection). The ratio of horizontal to vertical advection (α) is shown for the observations and the multi-model mean (MMM) above each panel. The α=1 and the α=-1 lines are shown. Points that lie to the right of the two lines (as in **a**) indicate that α>1 and horizontal temperature advection dominates over vertical temperature advection. Points that lie above the two lines (as in **c**) indicated that -1<α<1 and vertical temperature advection dominates over horizontal temperature advection. All available model ensemble members are shown. Figure details as per Fig. 2 of the manuscript.

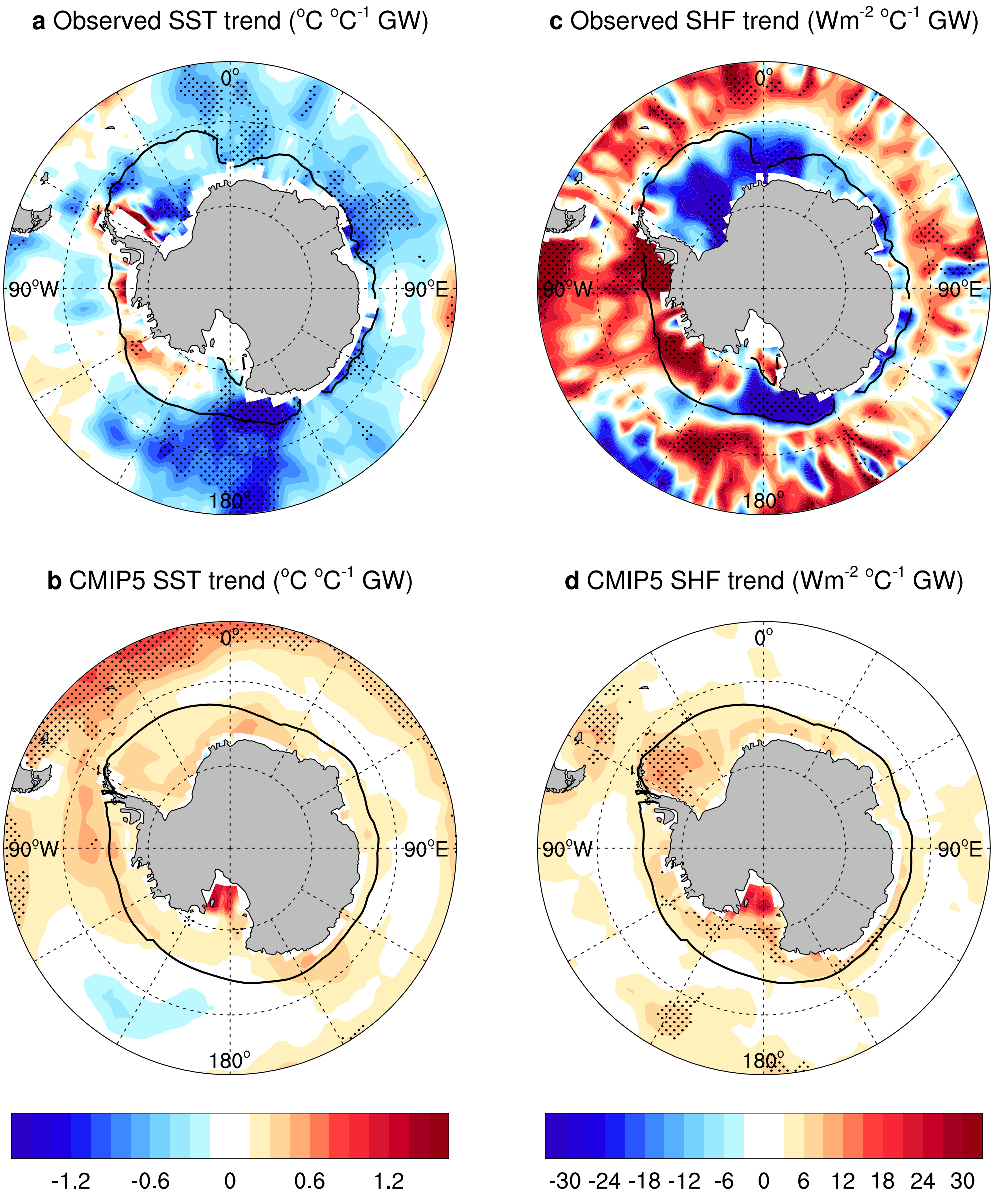

Supplementary Figure 6 | DJF trends in SST and downward total heat flux (positive heating the ocean) over 1979–2013. **a** Observed SST from HadISST, **b** CMIP5 multi-model mean SST, **c** observed heat flux from ERA-Interim, and **d** CMIP5 multi-model mean heat flux. Trends are expressed as a change per degree of global warming. Multi-model means are calculated using the first available ensemble member for each model. Stippling indicates significance: **a**, **c** at the 95% level as determined by a two-sided Student's *t* test, and **b**, **d** where 80% of models agree on the sign of the mean trend, which corresponds to 30 out of 37 models. The mean-state 15% SIC contour is shown in black.

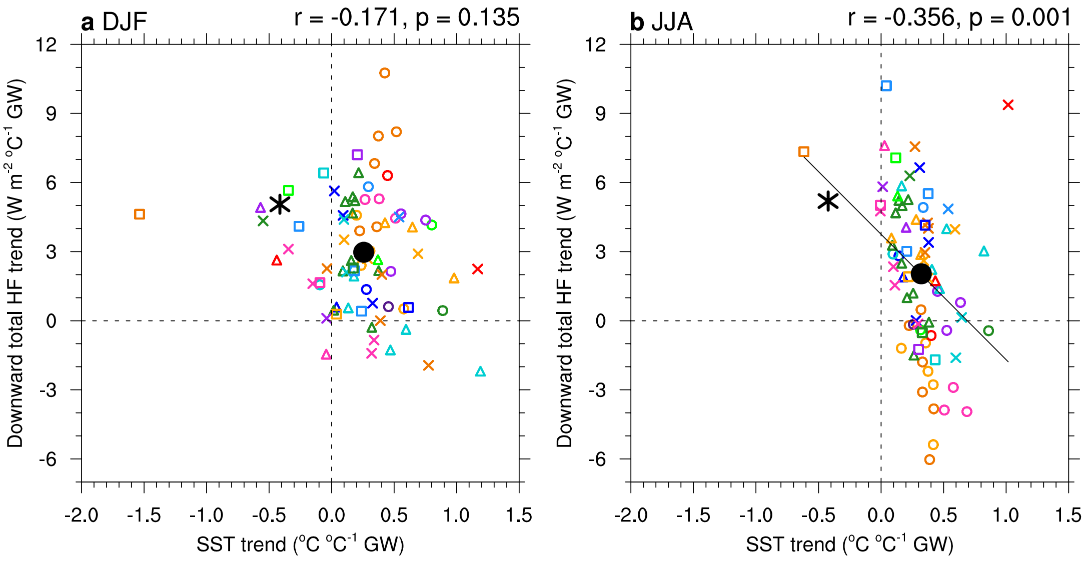

Supplementary Figure 7 | Trends in downward total heat flux (positive heating the ocean) versus SST over 1979–2013. a DJF, and **b** JJA. Trends are expressed as a change per degree of global warming and are calculated over 55–65°S. Observed heat flux from ERA-Interim, although considerable uncertainty exists in this estimate. Figure details as per Fig. 2 of the manuscript.

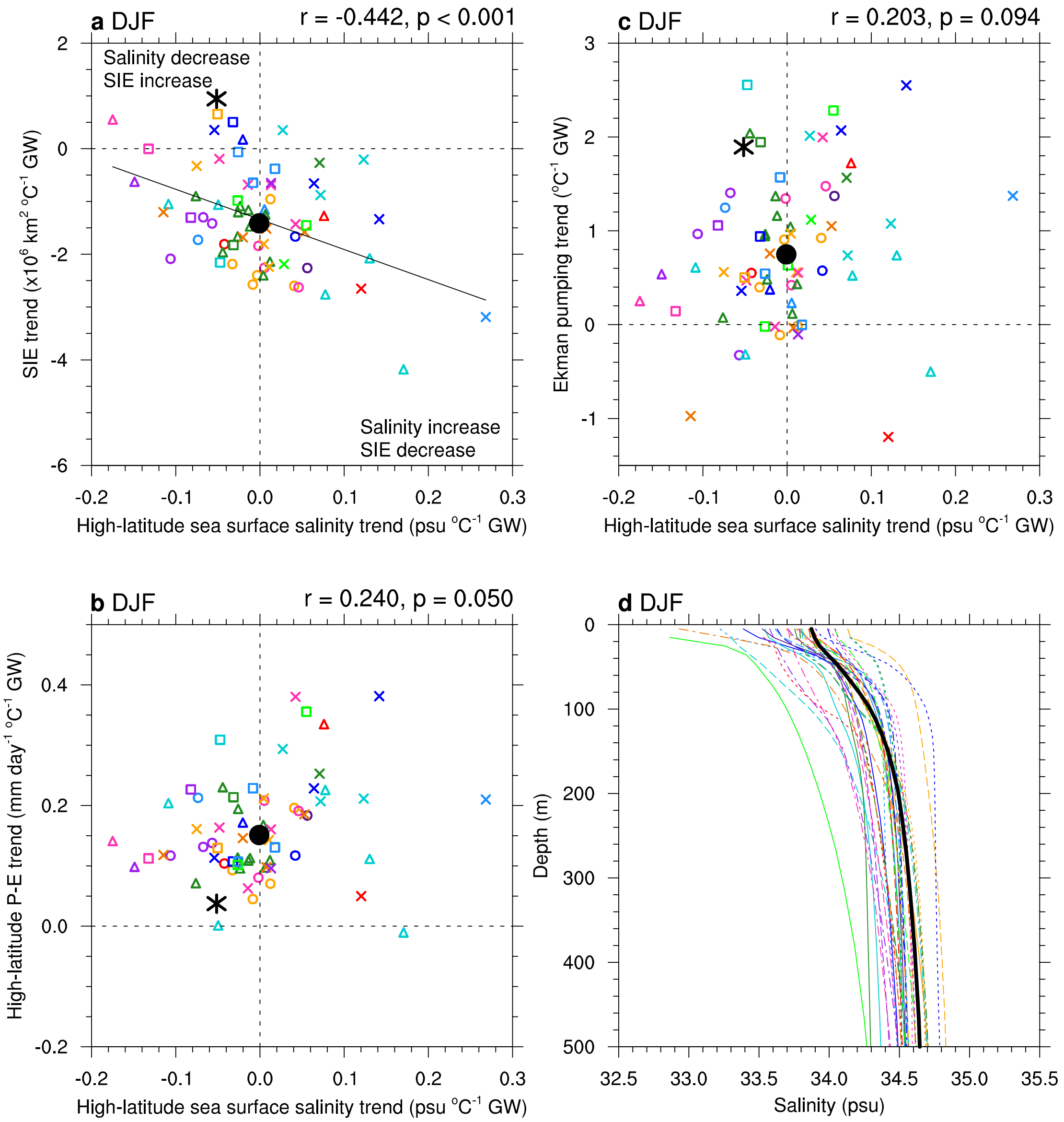

Supplementary Figure 8 | DJF trends over 1979–2013 and average salinity profiles over 1979–2005. **a** trends in SIE versus trends in high-latitude sea surface salinity, **b** trends in high-latitude ocean P–E versus trends in high-latitude sea surface salinity, **c** trends in Ekman pumping versus trends in high-latitude sea surface salinity, and **d** climatological zonal-mean salinity profiles averaged over 60–70°S. Trends are expressed as a change per degree of global warming. All available model ensemble members are shown in **a**–**c**. The first available ensemble member is shown in **d**. Observed salinity (black line in **d**) from SODA and Ishii, and precipitation from ERA-Interim. Ekman pumping trends in **c** are calculated as the trend in the Ekman pumping PC. Figure details for **a**–**c** as per Fig. 2 of the manuscript.

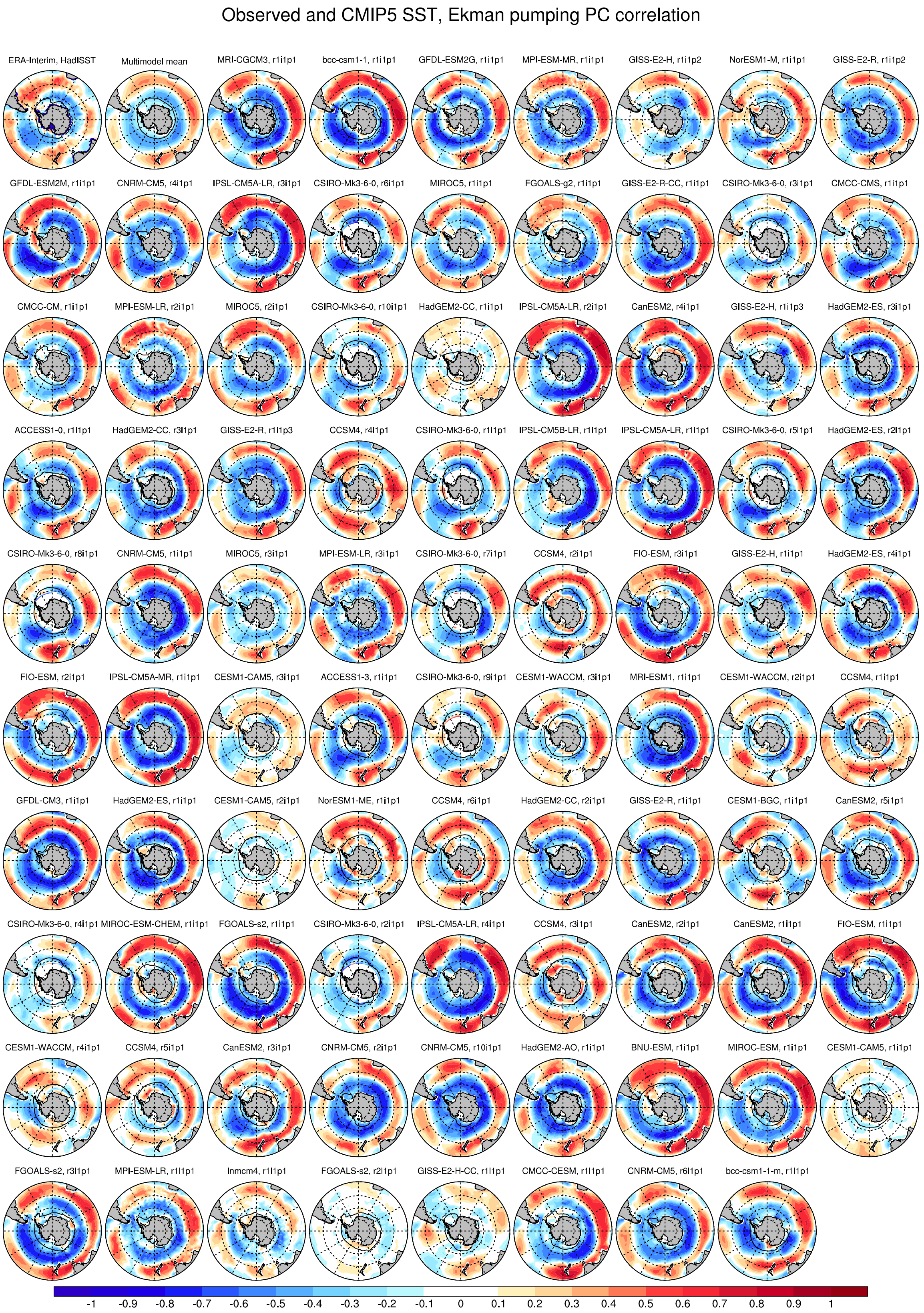

Supplementary Figure 9 | DJF correlations between SST and Ekman pumping PCs for observations and models over 1979–2013. Interannual correlations are calculated using linearly de-trended time series. (top left) Observed wind stress from ERA-Interim, SST from HadISST. (top second left) Multi-model mean. Individual models are ordered as per Supplementary Fig. 1.

Supplementary Table 1 | Inter-model correlation coefficients between trends over 1979–2013.

	DJF	*MAM*	*JJA*	*SON*
SIE–SST	**-0.70 (<0.001)**	**-0.75 (<0.001)**	**-0.70 (<0.001)**	**-0.73 (<0.001)**
Jet strength–SIE	**0.30 (0.004)**	**0.29 (0.006)**	0.18 (0.10)	0.10 (0.38)
Jet strength–SST	**-0.55 (<0.001)**	**-0.38 (<0.001)**	**-0.37 (<0.001)**	-0.18 (0.10)
Jet position–SIE	-0.06 (0.57)	-0.17 (0.12)	-0.09 (0.41)	0.05 (0.66)
Jet position–SST	**0.52 (<0.001)**	**0.29 (0.007)**	**0.28 (0.010)**	0.14 (0.20)
Ekman pumping–SIE	0.17 (0.13)	0.13 (0.25)	0.05 (0.64)	0.10 (0.37)
Ekman pumping–SST	**-0.54 (<0.001)**	-0.12 (0.30)	-0.03 (0.79)	-0.19 (0.087)
Ekman transport–SIE	0.03 (0.42)	0.16 (0.15)	0.16 (0.16)	-0.03 (0.82)
Ekman transport–SST	**-0.50 (<0.001)**	-0.21 (0.065)	**-0.28 (0.011)**	-0.03 (0.82)

Correlation coefficients between modelled trends in two metrics (p-values in brackets), calculated for all available model ensemble members. Correlations significant at the 95% confidence level are shown in bold. Jet position defined such that an equatorward trend is positive and a poleward trend is negative. Ekman pumping and Ekman transport trends are calculated as the trends in the Ekman PCs multiplied by the mean-state vertical and horizontal temperature gradients, respectively (as in Fig. 4, Supplementary Fig. 4).

Supplementary Table 2 | Interannual correlation coefficients between seasonal metrics over 1979–2013.

		DJF	*MAM*	*JJA*	*SON*
Jet strength–SIE	*Observed*	**0.39 (0.025)**	**0.35 (0.039)**	0.21 (0.23)	0.03 (0.87)
	CMIP5 median	0.16 (0.37)	0.18 (0.29)	0.06 (0.75)	0.10 (0.56)
Jet strength–SST	*Observed*	**-0.40 (0.016)**	-0.14 (0.43)	-0.01 (0.95)	-0.10 (0.58)
	CMIP5 median	**-0.47 (0.005)**	-0.21 (0.22)	-0.17 (0.33)	**-0.39 (0.020)**

Observed and median CMIP5 correlation coefficients (p-values in brackets). Significance takes into account the lag-1 autocorrelation. For the median CMIP5 correlation, the p-value is calculated using the median lag-1 autocorrelation. Correlations significant at the 95% confidence level are shown in bold. Individual CMIP5 correlation coefficients are shown in Supplementary Fig. 3.

Supplementary Table 3 | Additional inter-model correlation coefficients between trends over 1979–2013.

	DJF	*MAM*	*JJA*	*SON*
Evaporation–SST	**0.69 (<0.001)**	**0.76 (<0.001)**	**0.63 (<0.001)**	**0.51 (<0.001)**
Cloud cover–SST	-0.15 (0.16)	0.08 (0.48)	0.19 (0.076)	0.23 (0.033)
SIE–SSS	**-0.44 (<0.001)**	-0.11 (0.35)	**-0.37 (0.002)**	**-0.42 (<0.001)**
Ekman pumping–SSS	0.20 (0.094)	0.23 (0.058)	0.12 (0.34)	-0.02 (0.89)
P–E–SSS	0.24 (0.050)	0.15 (0.21)	0.11 (0.38)	0.00 (0.99)

As for Supplementary Table 1, for additional metrics assessed in the “Considering other mechanisms” section of the manuscript. SSS refers to sea surface salinity. Ekman pumping trends in this table are calculated as the trend in the Ekman pumping PC.
